# SATRAP: SOLiD Assembler TRAnslation Program

**DOI:** 10.1371/journal.pone.0137436

**Published:** 2015-09-14

**Authors:** Davide Campagna, Fabio Gasparini, Nicola Franchi, Lucia Manni, Andrea Telatin, Nicola Vitulo, Loriano Ballarin, Giorgio Valle

**Affiliations:** 1 CRIBI Biotechnology Centre, Università di Padova, Padova, Italy; 2 Department of Biology, Università di Padova, Padova, Italy; Georgia Institute of Technology, UNITED STATES

## Abstract

SOLiD DNA sequences are typically analyzed using a reference genome, while they are not recommended for *de novo* assembly of genomes or transcriptomes. This is mainly due to the difficulty in translating the SOLiD color-space data into normal base-space sequences. In fact, the nature of color-space is such that any misinterpreted color leads to a chain of further translation errors, producing totally wrong results. Here we describe SATRAP, a computer program designed to efficiently translate *de novo* assembled color-space sequences into a base-space format. The program was tested and validated using simulated and real transcriptomic data; its modularity allows an easy integration into more complex pipelines, such as Oases for RNA-seq *de novo* assembly. SATRAP is available at http://satrap.cribi.unipd.it, either as a multi-step pipeline incorporating several tools for RNA-seq assembly or as an individual module for use with the Oases package.

## Introduction

SOLiD DNA sequencers produce “color-space” reads, using 2-base encoding [1]. In essence, a sequence is represented as a series of transitions between adjacent bases. Since there are 16 combinations of 2 bases and there are only four available colors, each color represents four possible 2-base words, as shown in Table 1

**Table 1:** 2-base encoding table.

Color				
0	AA	CC	GG	TT
1	AC	CA	GT	TG
2	AG	CT	GA	TC
3	AT	CG	GC	TA

Colors are defined by numerical values (0, 1, 2, 3). Each color represents four possible dinucleotides.

For instance, the five base long sequence ACTAA is represented as 1230 because the encoding is AC = 1, CT = 2, TA = 3 and AA = 0, but it should be noticed that also the sequences CAGCC, GTCGG, TGATT would be represented by the same color sequence. However, the knowledge of the first base allows the selection of the right frame. Indeed, color space reads start from the last base of the adaptor, which is known, thus allowing the translation into bases. Therefore, in the given example, our read would be A1230 and the first color '1' will be translated into the first translated base (FTB) 'C' because AC corresponds to color 1. Potentially, reads could be entirely translated into bases, but any sequencing error would put the translation out of frame. Thus, while the first translated base is generally reliable, the risk of continuing with a completely wrong sequence increases progressively as we move away from the first position.

For the above reason, most SOLiD users do not translate color-space to base-space. Instead, it is preferable to translate the reference genome into color-space (which can be done unambiguously) and map the 2-base encoded reads on the color-space genome. The lack of a robust tool for translating colors into bases has hampered the use of SOLiD for *de novo* assembly applications. Some success has been obtained with the assembly of small genomes [2], using high coverage. In the case of RNA-seq data, where many transcripts are present at low levels, color translation is unfeasible with the software currently available.

*De novo* assembly of transcriptomes is in any case a complex process. Several programs have been developed for normal base-space reads, including Trinity [3] and Oases [4], but only Oases can be used to process color-space reads. Oases is based on the general assembler Velvet that is able to process SOLiD reads by “double encoding” [5]. Double encoding (DE) involves direct replacement of the four color symbols with A, C, G and T. The resulting sequence is still a color-space sequence, but since it uses the same symbols of normal DNA sequences, it can be processed by standard software. The main adaptation of Velvet for SOLiD sequences is related to kmer-counting, as color-space has a different strand complementarity.

Velvet and Oases do not implement color-space to base-space translation that until now could be done either with ASID (Applied Biosystems, unpublished) or SOPRA [6]. Both programs take advantage of the reliability of the base translated on the first color, giving satisfactory results only with high sequence coverage; therefore, they are not suitable for *de novo* transcriptome assembly. Here we present SATRAP, a color-space translation program designed to solve this problem. First SATRAP performs the analysis of color-space coherency, then it translates low covered transcripts with high accuracy, recognizing and correcting common types of assembly errors.

## Methods

SATRAP takes as input a double encoded assembly. The aforementioned Oases is ideal for transcriptome assembly, while Velvet or SSAKE [7] could be used for *de novo* genomic assembly. Then SATRAP takes full advantage of color-space to resolve conflicts due to incoherency in color assembly. Three main steps are implemented in the pipeline: 1) mapping FTBs onto the assembly; 2) analysis of color coherence; 3) error correction.

### Mapping FTBs onto the assembly

**Fig 1:**
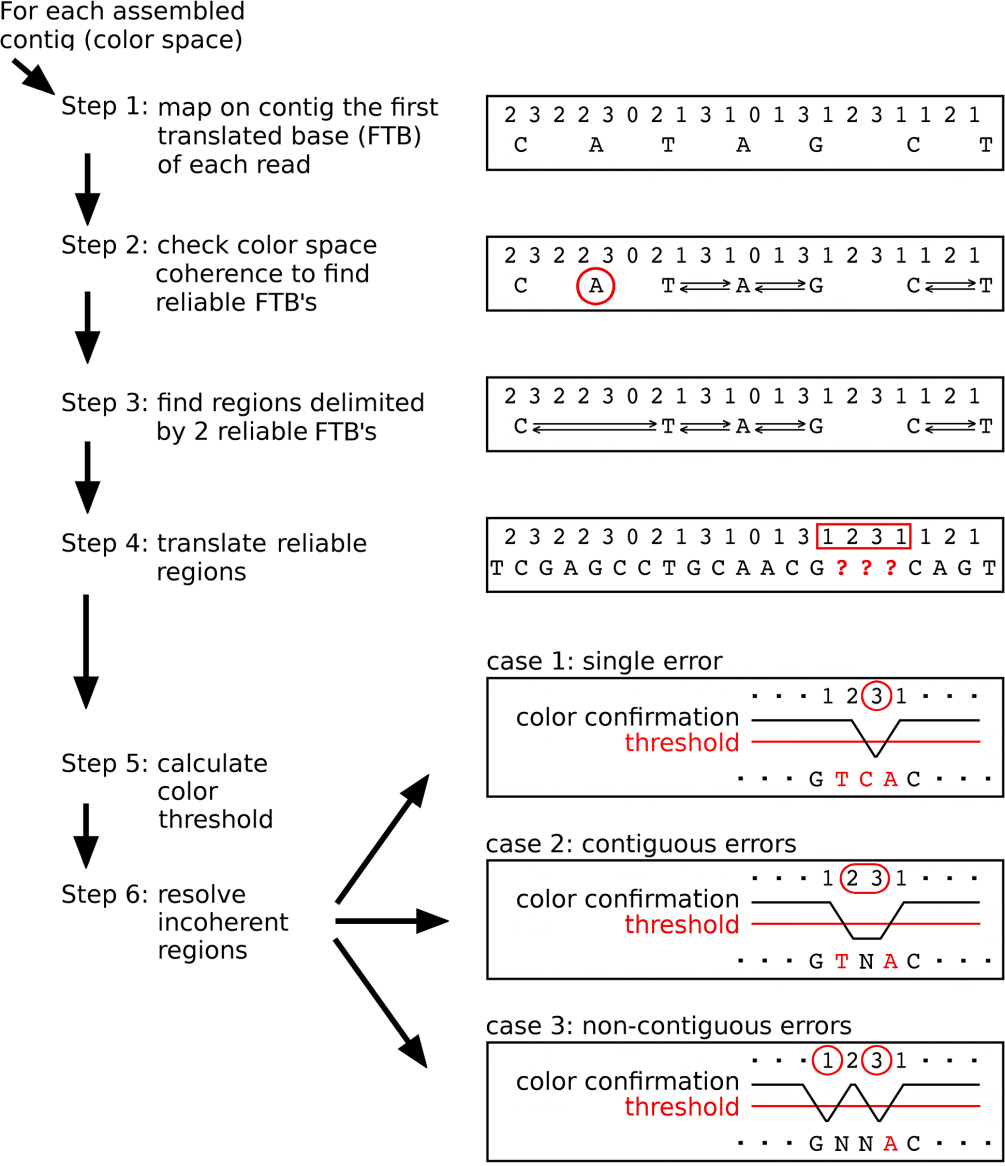
Flowchart of the color-translation process. Step1: the first base (FTB) of each read can be translated from color-space with high accuracy; for each read the FTB is mapped on the contig. Step 2: check color coherence with neighboring FTBs; three conditions can be detected: a) FTBs coherent with their neighboring FTBs on both sides (such as the 'A' at the centre of the figure); FTB coherent only on one side (such as the 'G' that is coherent with the 'A', but not with the 'C'); FTBs with no coherence on both sides (such as the 'A' circled in red). The latter are removed from the assembly. Step 3 and 4: find regions delimited by two reliable start sites and translate color-space into base-space. Any remaining regions will be incoherent in terms of color compatibility. To resolve these regions the threshold for color reliability is calculated (Step 5) and the resulting value is used to establish the critical regions of the contig (Step 6).

This process requires multiple steps because the first color of the read depends on the last base of the adaptor and may not align on the target. For this reason, mapping algorithms for SOLiD reads ignore the first color and align the reads starting from the second color. Therefore, in the resulting alignment, the first color is missing, thus complicating the recovery of the FTB. To allow the retrieval of FTBs, the reads are aligned on the assembled transcripts using a special option of PASS [8] that includes the FTB information in the read name of each alignment. Finally, Satrap uses this information to place the FTB just before the color alignment (Fig 1, Step 1).

### Analysis of color coherence

We define as reliable regions those which are delimited by two adjacent FTBs joined by a coherent stretch of colors (double arrows in Fig 1). If more FTBs map on the same position then the most frequent base will be considered. After a first scan (Fig 1, Step 2) some FTBs may have unreliable regions on both sides (Fig 1, 'A' in the red circle). Such FTBs will be discarded and the new resulting regions will be reconsidered for color coherence (Fig 1, Step 3). All the reliable regions are then translated from colors to bases (Fig 1, Step 4). The remaining regions (shown as question marks in the figure) are not coherent with the sequence of colors, but are delimited by two reliable regions. For those cases an internal error correction is possible.

### Error correction

The error correction is completing the SATRAP pipeline (Fig 1, Steps 5 and 6). The regions enclosed within FTBs that are not coherent with the color sequence are selected and analyzed in detail. In a position corresponding to an assembly error we expect a lower percentage of matched colors. For each position enclosed within FTBs, the number of colors that match with the reference is compared to a threshold (1) specifically calculated for each transcript.

T=Max(0,M−Z⋅σ) (1)

In the above equation T is the threshold to discriminate unreliable colors, M is the mean of matched colors confirming the consensus of each position, σ is the standard deviation of M and Z is the set Z-score.

Once the quality threshold is calculated (Fig 1, Step 5) it can be applied to discriminate unreliable colors (Fig 1, step 6). When only one color is below threshold the two related bases can be inferred from the adjacent colors and a full correction can be achieved (Fig 1, case 1). If more colors are found below threshold then the entire region enclosed between the unreliable colors will be translated into N's (Fig 1, case 2 and 3).

## Results and Discussion

**Fig 2:**
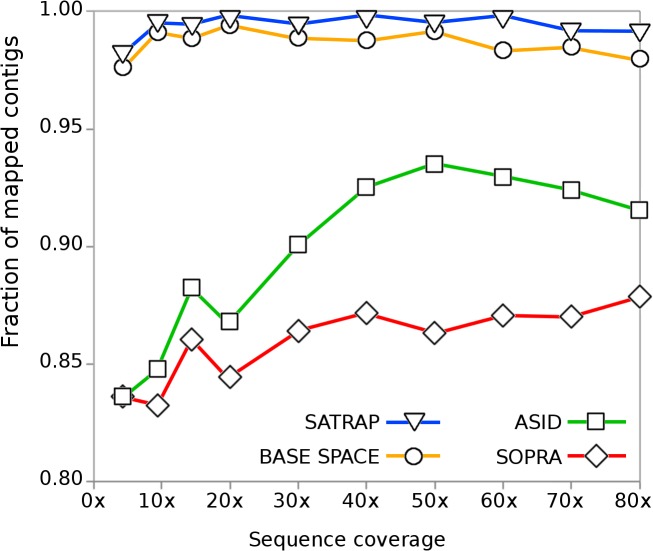
Effect of sequence coverage on color translation. ASID, SATRAP and SOPRA were used to translate the color-space assemblies produced at different sequence coverage into base-space. The same set of reads was also assembled in base-space as a control.

The validation of the program was done both with simulated and real datasets. To assess how the sequence coverage affects the translation process we used dwgsim (https://github.com/nh13/) to generate simulated reads (S1 Text) that were assembled with Velvet (S2 Text) and then color-translated with SATRAP, ASID and SOPRA (S3 Text). To make it more critical, the reads were generated with a relatively high rate of sequencing errors (from 0.001 to 0.2 linearly applied to the base positions of each read) and mutations (0.03) that include 10% of insertions and deletions. To generate the simulated set of reads we took the transcriptome of *Ciona intestinalis*, composed by 15,852 transcripts. A base-space assembly was also processed as a positive control, using a set of reads corresponding to those in color-space. The fraction of translated assemblies that entirely mapped (S4 Text) onto the *C*. *intestinalis* reference transcripts are shown in Fig 2.

SATRAP was also tested for its ability to recognize specific assembly errors. The simulation was performed using 1,000 random base-space sequences with a size range between 200 and 650 bases. The base-space sequences were used to produce a data set of simulated color-space reads that had a sequence coverage up to 100X. The 1,000 random base-space sequences were converted to color-space through reverse 2-base encoding and then a single error was inserted in the middle of each color sequence. One thousand errors were introduced: 334 substitutions, 344 deletions and 322 insertions (S5 Text). These parameters make this simulation particularly challenging for color translation because about 2/3 of errors are indels. After translation, the subsequent base-space sequences were globally aligned onto the original error-free base-space dataset using PASS (S6 Text). The results shown in Table 2 were obtained with Z = 3 and indicate that there is a very high efficiency of translation at all coverages.

**Table 2:** Statistics of identified errors at different sequence coverage.

Coverage	Substitution	Deletion	Insertion
10X	1	0.988	0.994
20X	1	0.988	0.997
50X	1	1	1
100X	1	0.997	0.997

Finally, SATRAP was evaluated on a Human RNA-seq set of data publicly available (ERR200630). The reads were assembled using Oases and then translated using SATRAP (S7 Text). As expected, the number of FTBs with unreliable regions on both sides was extremely low (997 out of 9,657,411 FTBs), indicating very high accuracy in FTB translation. The majority of corrections were related to single color errors (85%) and at the end of the process SATRAP corrected 485,391 bases while 47,424 bases remained undefined (N's). As a result, 95.6% of translated contigs totally mapped on the reference Human genome. Furthermore, about 29.2% of non-mapping contigs mapped on the non-redundant sequence database using Blastx [9]. These results compare quite well with the Illumina RNA-seq run (SRR090440) where 95.4% of the reads mapped on the reference genome and 1.9% of the remaining sequences mapped on the nr database.

## Conclusion

The translation of the ERR200630 assembly required less than 1 Gb of RAM and one hour of elaboration time using a 64 bits Intel Xeon E5645 CPU working at 2.4 GHz. It is notable that the total time spent by Satrap is only a small fraction of that taken by the assembly process.

All the computer programs are written in the C++ language and released into public domain under the GNU general public license.

The results presented in this paper show that, with SATRAP, the final color assembly converted into bases is essentially equivalent to those obtained with base-space reads. We conclude that it is now possible to achieve *de novo* assembly of color-space reads as efficiently as using base-space.

## Supporting Information

S1 Text SOPRA, SATRAP and Asid comparison: simulated datasets. Information regarding the production of the simulated dataset (setting and criteria) for SOPRA, SATRAP and Asid comparison.
(PDF)

S2 Text SOPRA, SATRAP and Asid comparison: simulated assemblies. Setting information about the assembly of the simulated dataset reported in S1.
(PDF)

S3 Text SOPRA, SATRAP and Asid comparison: translation setting. Information about the setting of SOPRA, SATRAP and Asid programs for the translation of the simulated assemblies.
(PDF)

S4 Text SOPRA, SATRAP and Asid comparison: comparison of results. Mapping information of the translated assemblies reported in Fig 2.
(PDF)

S5 Text Evaluation of SATRAP to identify different assembly errors: dataset production. Information about programs and setting to produce the simulated dataset for the assembly error detection.
(PDF)

S6 Text Evaluation of SATRAP to identify different assembly errors: translation and global mapping. Information regarding both translation and mapping of simulated data that are described in S5. The mapping information is used to calculate the information reported in Table 1.
(PDF)

S7 Text Evaluation of SOLiD and ILLUMINA RNA-seq assemblies. Comparison of real base-space and color-space translated assemblies in terms of total mappable contigs.
(PDF)
