# Supplementary material for: SATRAP: SOLiD Assembler TRAnslation Program
Source: PLoS One. 2015 Sep 14;10(9):e0137436. doi: 10.1371/journal.pone.0137436 (PMC4569514; doi:10.1371/journal.pone.0137436)
Supplement: S1 Text — Information regarding the production of the simulated dataset (setting and criteria) for SOPRA, SATRAP and Asid comparison. (PDF) [file pone.0137436.s001.pdf]

SOPRA, SATRAP and Asid comparison: simulated datasets

The color-space dataset of simulated reads was produced using the Ciona intestinalis predicted transcripts (Dehal et al. 2002. Science 298, 2157-2167). Referring to the data release policy of C. intestinalis sequence information, this sentence must be reported: "these sequence data were produced by the US Department of Energy Joint Genome Institute http://www.jgi.doe.gov/ ".

Using the C. intestinalis transcripts as reference, 10 datasets of simulated color- space reads were generated at the following sequence coverage: 5X, 10X, 15X, 20X, 30X, 40X, 50X, 60X, 70X, 80X using the dwgsim-0.1.8 program available at:
http://sourceforge.net/apps/mediawiki/dnaa/index.php?title=Whole_Genome_Simulation

In this analysis the transcript isoforms with size < 300 bases were not considered.

dwgsim setting

Simulated reads production

```
dwgsim -y 0 -z 0 -d 100 -S 2 -c 1 -1 76 -2 35 -C $cov \
-e 0.001-0.2 -E 0.001-0.2 -r 0.03 -R 0.1 \
SIMULATION/great_300.fa \
SIMULATION/tcolor.fasta
```

The per base/color/flow error rate was set respectively to 0.001-0.2 linearly applied to the read size. The rate of mutation was set to 0.03 (10% of these mutations were indels). Finally, "-C" allows to set the mean coverage ($cov) across the available positions of the template reference.

Our control dataset is constituted by simulated reads (base-space) that exactly map in the same position of the color-space ones (reported in figure 2 of the manuscript). To obtain this dataset the parameter "-c" was set to 0.
