# Supplementary material for: SATRAP: SOLiD Assembler TRAnslation Program
Source: PLoS One. 2015 Sep 14;10(9):e0137436. doi: 10.1371/journal.pone.0137436 (PMC4569514; doi:10.1371/journal.pone.0137436)
Supplement: S2 Text — Setting information about the assembly of the simulated dataset reported in S1. (PDF) [file pone.0137436.s002.pdf]

SOPRA, SATRAP and Asid comparison: simulated assemblies

For each considered coverage, the simulated color-space reads were double-encoded using the program "2csfastq_1csfastq" inside the SATRAP pipeline and then assembled using the Velvet program. The setting information is described below:

Double encoding setting

```
2csfastq_1csfastq \
-csfastq1 color.read1.fastq \        # first file of color-space simulated reads
-csfastq2 color.read2.fastq \        # second file of color-space simulated reads
-tags "/2" "/1" \                    # tags inside the read name
-q 1 \                               # Quality filter. It Considers all reads with quality >= 1
-double-encoded \                    # parameter to enable the double-encoding
> DE.fastq                           # Output file
```

velvelh_de setting

```
bin/velveth_de VELVET_ASSEMBLY/  27 -short fastq DE.fastq
```

The following variables were set as a function of sequence coverage ($COVERAGE) and then applied to set the velvetg program.

$MAX_COVERAGE= ($COVERAGE+ $COVERAGE/ 2)
$CUTOFF=$COV - $COV / 5

velvelg_de setting

```
velvetg_de VELVET_ASSEMBLY/ -read_trkg yes -amos_file yes -cov_cutoff  $CUTOFF \
-min_contig_lgth 100 -exp_cov $COVERAGE -max_coverage $MAX_COVERAGE \
-long_mult_cutoff $CUTOFF -max_divergence 0.05
```

Please, see the Vevet manual for details about the parameters. The resulting assemblies were translated using SATRAP, SOPRA and Asid programs.
