# Supplementary material for: SATRAP: SOLiD Assembler TRAnslation Program
Source: PLoS One. 2015 Sep 14;10(9):e0137436. doi: 10.1371/journal.pone.0137436 (PMC4569514; doi:10.1371/journal.pone.0137436)
Supplement: S3 Text — Information about the setting of SOPRA, SATRAP and Asid programs for the translation of the simulated assemblies. (PDF) [file pone.0137436.s003.pdf]

SOPRA, SATRAP and Asid comparison: translation setting

Please, see the manual of SOPRA, SATRAP and Asid for details about the used parameters.

SOPRA color translation setting

```
 perl  solid_denovo_preprocessor.pl \
-file color.read1.fastq.csfasta \
-file color.read2.fastq.csfasta

 perl  solid_denovo_postprocessor.pl \
-csfasta output/colorspace_input.csfasta \
-afgfile VELVET_ASSEMBLY/velvet_asm.afg \
-output VELVET_ASSEMBLY/contigs.bs

 perl format_col_v1.4.6.pl \
-frag color.read1.fastq.csfasta  \
color.read2.fastq.csfasta \
-a output/

perl vs_contig_col_v1.4.6.pl \
-f VELVET_ASSEMBLY/contigs.bs \
-a output/
```

SATRAP color translation setting

This setting regards the specific programs managed by SATRAP pipeline.

```
pass \
-cpu 8 -double_encoded \
-d VELVET_ASSEMBLY/contigs.fa \
-fastq DE.fa \
-g 3 -fle 25 -l -fid 90 -sam -query_size 200 -b \
-pst_word_range 6 6 \
> output/DE.sam \
2> output/DE.log

cs2bs_assembly \
-fasta VELVET_ASSEMBLY/contigs.fa \
-sam output/DE.sam \
-l 30 -n 0.1 -z 3 -erode 3 \
> output/BS_contig.fa \
2>output/BS_contig.log
```

Asid 1.0 color translation setting

```
denovo2/utils/solid_denovo_preprocessor_v1.2.pl \
--run_type fragment --output assembly/preprocessor \
--f3_file simulated_solids_reads.csfasta

denovo2/velvet_1.2.10/velveth_de \
assembly/velvet 27 -fasta \
-short assembly/preprocessor/doubleEncoded_input.de

denovo2/asid.1.0/asid_light \
-merge assembly/velvet/LastGraph assembly/velvet/contigs.fa \
assembly/preprocessor/colorspace_input.csfasta \
assembly/postprocessor/colorspace_input.idx \
```

```
assembly/postprocessor/gap_reads/ \
assembly/postprocessor/color_reads.ma \
assembly/asid_scaffolds.de \
graph2ma 100 fragment

denovo2/asid.1.0/asid_light \
-convert assembly/postprocessor/color_reads.ma 70 \
 > assembly/nt_contigs.fa
```
