# Supplementary material for: SATRAP: SOLiD Assembler TRAnslation Program
Source: PLoS One. 2015 Sep 14;10(9):e0137436. doi: 10.1371/journal.pone.0137436 (PMC4569514; doi:10.1371/journal.pone.0137436)
Supplement: S4 Text — Mapping information of the translated assemblies reported in Fig 2. (PDF) [file pone.0137436.s004.pdf]

SOPRA, SATRAP and Asid comparison: comparison of results

This analysis was performed to discriminate badly assembled contigs (for instance chimeric contigs coming from the assembling of two or more different transcripts) by badly translated contigs. Firstly, we performed a global mapping of the translated assemblies onto the C. intestinalis transcripts, then the remaining not aligned contigs were locally mapped onto the same transcriptome.

We considered the not aligned contigs mapped for at least 90% of their sequence length. This information was added to the number of global mapped contings and reported in figure 2 of the manuscript.

Global mapping setting using PASS program

```
 pass \
-fasta BS_contig.fa \
-d reference_transcripts.fa \
-fid 90 -query_size 20000 \
-p 1111110111111 -block 1000 \
-check_block 1000 -sam -g 3 -cpu 8 \
-pst_word_range 6 6  \
-not_aligned \
>/dev/null \
2>RESULT.log                                        # used for statistics
```

Local mapping setting using PASS program

```
 pass \
-fasta not_aligned.fa \
-d  reference_transcripts.fa \
-fid 90 -query_size 20000 -p 1111110111111 \
-p 1111110111111 -block 1000 \
-check_block 1000 -sam -g 3 -cpu 8 \
-pst_word_range 6 6  -l -fle 40 \
>local_aligned.sam \
2>local_aligned.log
```

Recovering contigs using local mapping results

```
fraction_aligned \
-fasta not_aligned.fa \                          # file containing the not aligned contigs
-sam local_aligned.sam \                         #  file containing the local alignments
-thr 0.9 \                                       #  Fraction of sequence length threshold
>recovered_statistics.txt                        # Statistics of recovered contigs
```

The program “fraction_aligned” was specifically developed to calculate the statistics of local mapped reads. Please, see the manual of PASS program (http://pass.cribi.unipd.it) for details about the setting.
