# Supplementary material for: SATRAP: SOLiD Assembler TRAnslation Program
Source: PLoS One. 2015 Sep 14;10(9):e0137436. doi: 10.1371/journal.pone.0137436 (PMC4569514; doi:10.1371/journal.pone.0137436)
Supplement: S5 Text — Information about programs and setting to produce the simulated dataset for the assembly error detection. (PDF) [file pone.0137436.s005.pdf]

Evaluation of SATRAP to identify different assembly errors: data set production

Firstly, we have generated 1000 random base space sequences with a range of size between 200 to 650 bases in length. After that, these sequences were double-encoded and 1 assembly error was inserted at the middle of each color space sequence: 334 substitutions, 344 deletions and 322 insertions were considered. The 1000 random base space sequences were passed as template for dwgsim-0.1.8 program to generate 4 dataset of simulated color space reads. A total of 4 analyses were considered; one for each coverage value (10X, 20X, 50X and 100X) reported in the setting as the variable $COVERAGE.

Setting of dwgsim program

```
dwgsim -y 0 -z 0 -d 0 -S 2 -c 1 -1 50 -2 0 -C $COVERAGE  -r 0  \
1000_base_space.fa \
COLOR_
```

Setting for double encoding

```
2csfastq_1csfastq \
-csfastq2 READS/COLOR_$COVERAGE.read1.fastq -fragment \
-tags /2 /1 -trim3 0 > READS/de_$COVERAGE.csfastq
```

Setting for double encoded reads mapping

```
pass -cpu 12 \
-double_encoded -g 3 \
-d 1000_color_space.fa \
-fastq READS/de_$COVERAGE.csfastq \
-fid 90 -sam -query_size 100 -b
> de_$COVERAGE.sam
2> mapped.log
```

Please, see the manuals of PASS (http://pass.cribi.unipd.it), SATRAP (http://satrap.cribi.unipd.it) and dwgsim (http://sourceforge.net/apps/mediawiki/dnaa/index.phptitle=Whole_Genome_Simulation) for detailed information about the parameters.
