# Supplementary material for: SATRAP: SOLiD Assembler TRAnslation Program
Source: PLoS One. 2015 Sep 14;10(9):e0137436. doi: 10.1371/journal.pone.0137436 (PMC4569514; doi:10.1371/journal.pone.0137436)
Supplement: S6 Text — Information regarding both translation and mapping of simulated data that are described in S5. The mapping information is used to calculate the information reported in Table 1. (PDF) [file pone.0137436.s006.pdf]

Evaluation of SATRAP to identify different assembly errors: translation and global mapping

The translated assemblies were globally mapped onto the original base space sequences to infer the statistics of translation. Number and kind of errors are then analyzed and reported in table 1 of the manuscript.

Setting for Color space translation

```
cs2bs_assembly -fasta 1000_color_space.fa \
-sam de_$COVERAGE.sam  -l 50 -n 0.1 -z 3 \
 -erode 3 > TRANSLATED.fa
```

Global mapping setting

```
 pass \
-fasta output/BS_contig.fa \
-d SIMULATION/TRANSLATED.fa \
-fid 90 -query_size 20000 \
-p 1111110111111 -block 1000 \
-check_block 1000 -sam -g 3 -cpu 8 \
-pst_word_range 6 6  \
-not_aligned \
>/dev/null \
2>RESULT.log
```

Please, see the user manual of PASS and SATRAP for further information about the parameters.
