# Supplementary material for: SATRAP: SOLiD Assembler TRAnslation Program
Source: PLoS One. 2015 Sep 14;10(9):e0137436. doi: 10.1371/journal.pone.0137436 (PMC4569514; doi:10.1371/journal.pone.0137436)
Supplement: S7 Text — Comparison of real base-space and color-space translated assemblies in terms of total mappable contigs. (PDF) [file pone.0137436.s007.pdf]

Evaluation of SOLiD and ILLUMINA RNA-seq assemblies

The RNA-seq runs ERR200630 and SRR090440 were sequenced respectively using the AB 5500 Genetic Analyzer sequencer and the Illumina Genome Analyzer II sequencer. These runs were downloaded from the DDBJ SRA archive (http://trace.ddbj.nig.ac.jp/dra/index_e.html).
A de novo transcriptome assembly of ERR200630 was produced using the SATRAP pipeline that integrates both Oases and Velvet programs. Finally, the assembly ERR200630 was translated into base-space.
Except the double encoding and the color space translation steps, the ILLUMINA reads were assembled using the same setting that has been applied to the SOLID data.

Setting of the SATRAP pipeline

```
SATRAP/bin/satrap -step 1 2 3 4 \
-reads_path SOLID_DATASET/ \
-file_esten .fastq \
-tags _F3 _F5-RNA \
-tmp_dir RESULTS/ \
-velvet_path velvet_path/ \
-oases_path oases_path/ \
-q 18 -t1 5 -t2 0
```

The SOLiD reads of 50 bases in size were trimmed by 5 bases (-t1 5) while no trimming was applied for the ILLUMINA reads. For both SOLiD and ILLUMINA datasets, the reads were selected for the minimal mean base quality of 18. Finally, the multiple-kmer assemblies were assembled using the following kmer size: 21, 23, 25, 27, and 31 bases.
The assembled transcripts were firstly mapped using SPLIGN and COMPART programs (http://www.ncbi.nlm.nih.gov/sutils/splign/splign.cgi) with the default setting. Next, the not aligned contigs obtained from SPLIGN mapping were mapped into the nr protein database (ftp://ftp.ncbi.nih.gov/blast/db/FASTA/nr.gz) using blastx from BLAST+ (ftp://ftp.ncbi.nlm.nih.gov/blast/executables/blast+/LATEST/).

Results were analyzed considering the following criteria:
1) In the case of SPLIGN results, only the contigs that mapped entirely onto the hg19 genome were considered (the program "splign_analyze" was specifically developed to produce the statistics of mapped contigs).
2) Regarding the analysis inherent the blastx program, we used the following criteria to select the aligned contigs: i) the alignments must be representative of at least 90% of the contig length; ii) the e-value of the alignments must be lower or equal to 10-3, iii) the sequence identity of the alignments must be at least 90%.

Setting of SPLIGN program

It is used the same setting described in the manual of SPLIGN program (http://www.ncbi.nlm.nih.gov/sutils/splign/splign.cgi?textpage=documentation)
